# Prevalence of Atrophic Gastritis in Kazakhstan and the Accuracy of Pepsinogen Tests to Detect Gastric Mucosal Atrophy

**DOI:** 10.31557/APJCP.2019.20.12.3825

**Published:** 2019

**Authors:** Linda Mezmale, Sergejs Isajevs, Inga Bogdanova, Inese Polaka, Anna Krigere, Dace Rudzite, Aiga Rudule, Ilze Kikuste, Sergei Parshutin, Altynbek Tazhedinov, Dmitry Mushinskiy, Darkhan Sametayev, Tatyana Belikhina, Nurbek Igissinov, Jin Young Park, Rolando Herrero, Marcis Leja

**Affiliations:** 1 *Institute of Clinical and Preventive Medicine, *; 2 *Faculty of Medicine, University of Latvia, *; 3 *Academic Histology Laboratory, *; 4 *Digestive Disease Centre GASTRO, Riga, Latvia, *; 5 *Regional Diagnostic Centre, *; 6 *Central City Clinical Hospital, Almaty, *; 7 *Semey Regional Oncology Centre, Semey, *; 8 *Astana Medical University, *; 9 *Central Asian Cancer Institute, Nur-Sultan, Kazakhstan, *; 10 *International High School of Medicine, *; 11 *Eurasian Institute For Cancer Research, Bishkek, Kyrgyzstan, *; 12 *Prevention and Implementation Group, Section of Early Detection and Prevention, International Agency for Research on Cancer, Lyon, France. *

**Keywords:** Pepsinogens, atrophy, gastric cancer, atrophic, gastritis, Kazakhstan, gastric, screening

## Abstract

**Background:**

Atrophic gastritis is considered precursor condition for gastric cancer. There is so far limited evidence on the performance of pepsinogens for atrophy detection in Central Asia. The aim of our study was to detect the prevalence of atrophic gastritis in the asymptomatic adult population in Kazakhstan as well as address the accuracy of pepsinogen testing in atrophy detection.

**Methods:**

Healthy individuals aged 40-64 were included. Upper endoscopy and pepsinogens (PG) evaluation were performed. PG were analysed in plasma by latex agglutination. Cut off values were used to define decreased PG values (PGR ≤ 3 and PG I ≤ 70 ng/mL); severely decreased PG values (PGR ≤ 2 and PG I ≤ 30 ng/mL). Biopsies were analyzed and obtained according to the updated Sydney System. PG test sensitivity, specificity and overall accuracy were assessed using the histological diagnosis as the “gold standard”.

**Results:**

Altogether 157 individuals - female 40,1% and male 59,9% were included. Histologically, moderate to severe corpus atrophy, was present only in 1,3% cases. From all study subjects, 26,8% had decreased plasma PG values with cut-off values PGR ≤ 3 and PG I ≤ 70 ng/mL. The sensitivity of the PG test with this cut-off values was 50,0%, specificity 73,5%, overall accuracy 73,2% for detection of moderate to severe atrophy in the corpus. The sensitivity of PG test with cut-off values PGR ≤ 2 and PG I ≤30 ng/mL was 50,0%, specificity 90,9% and overall accuracy 90,4%.

**Conclusions:**

The prevalence of gastric mucosal atrophy was low in the Kazakh population. Serological PG test screening nevertheless can play an important role in the diagnosis of gastric precancerous lesions. However, the diagnostic accuracy of the PG test is mainly dependent on the cut-off values for positive results.

## Introduction

Gastric atrophy and intestinal metaplasia are considered as precancerous lesions in the stomach (Rugge et al., 2013; Choi et al., 2018); therefore, in high-gastric cancer risk countries, one would expect the increased prevalence of the lesions in the general population (Pimentel-Nunes et al., 2019). Surveillance of patients with these lesions may, therefore, result in early detection of gastric malignancy and improved prognosis (Rugge et al., 2010; Rugge et al., 2011; Pimentel-Nunes et al., 2019). Current management of epithelial precancerous conditions and lesions in the stomach guidelines recommended endoscopic surveillance and guided biopsies every three years (within three year period) in patients with advanced stages of atrophic gastritis (Pimentel-Nunes et al., 2019). 

The prevalence of precancerous lesions in the general population is known to vary around the globe, mostly depending on *Helicobacter pylori *(*H. pylori*) status. Most of the studies have reported atrophy below 50%, but in others from Asia, the prevalence is higher (Wong et al., 2004; Weck and Brenner, 2006; de Vries et al., 2008; Sonnenberg et al., 2010; Isajevs et al., 2014). Prevalence of gastric cancer is particularly high in Asia region and incidence varies according to the geographical area of Asia. Kazakhstan is a country in Central Asia with high gastric cancer burden. About three thousand new cases of gastric cancer in 2018 were registered in the Kazakhstan Republic, and it was the third most common cancer in this country (Bray et al., 2018). However, recent data show that gastric cancer dynamics of incidence and mortality revealed a downward trend. It is not consistent with global statistics, where forecast indicators are rising (Igissinov et al., 2018; Igissinov et al., 2019). 

Early detection of atrophic gastritis may reduce the incidence of gastric cancer. Nowadays, serum pepsinogens are considered the best available non-invasive method for gastric corpus atrophy detection (Agreus et al., 2012; Malfertheiner et al., 2017). 

There is so far limited evidence on the performance of pepsinogens for atrophy detection in Central Asian populations, as to our knowledge, the only studies that have been performed were using ELISA tests for pepsinogen detection (Benberin et al., 2013). The results of our group have confirmed the good correlation between pepsinogen measurements by ELISA and latex agglutination tests (Leja et al., 2017). 

The current study was aimed to detect the prevalence of atrophic gastritis in the asymptomatic adult population in Kazakhstan as well as address the accuracy of pepsinogen testing in atrophy detection.

**Table 1:** Histologically Detected Atrophy of Gastric Mucosa in Certain Parts of the Stomach

Part of the stomach	Mild atrophy, n (%)	Moderate atrophy, n (%)	Severe atrophy, n (%)
Antrum	120 (76,43)	12 (7,64)	0 (0)
Corpus	133 (84,71)	2 (1,27)	0 (0)
Incisura angularis	103 (65,61)	8 (5,10)	1 (0,64)

**Table 2:** Accuracy of Serum Pepsinogen Test with Different Cut-Off Values for Diagnosis of Corpus Atrophy

PGI, PGI/II cut-off values, %		
	PGI ≤ 70 ng/mL un PGI/II ≤ 3	PGI ≤ 30 ng/mL un PGI/II ≤ 2
Sensitivity (95% CI)	50.0 (1.2 - 98.7)	50.0 (1.2 - 98.7)
Specificity (95% CI)	73.5 (65.8 - 80.3)	90.9 (85.3 - 94.9)
PPV (95% CI)	2.3 (0.5 - 9.0)	6.7 (1.6 - 23.7)
NPV (95% CI)	99.1 (96.6 - 99.7)	99.3 (97.2 - 99.8)
Overall accuracy	73.2 (65.6 - 79.9)	90.4 (84.7 - 94.5)

PG, pepsinogen; PPV, positive predictive value; NNP, negative predictive value; 95% CI, 95% confidence interval.

## Materials and Methods

*Study design*

The study was conducted as part of the regional pilot study of GISTAR (*Gastric Cancer Prevention Study, *available at *https://www.gistar.eu/*) in collaboration with the project “Decreasing the burden of gastric cancer in Kazakhstan: evaluation of the existing situation and search for improvement possibilities” (AP05133849). The methodology applied for this project was developed by International Agency Research on Cancer (World Health Organization). The main aim of the GISTAR project is to find new strategies to decrease mortality from gastric cancer in high-risk areas (Leja et al., 2017). 

Therefore the pilot study was carried out in Kazakhstan – a country with a high incidence of gastric cancer. Three regional pilot research centres were opened in Kazakhstan – two in Almaty and one in Semey. Healthy volunteers were invited to participate in the pilot study. Detailed standard GISTAR questionnaire was administered to the study subjects, and the responses were entered into the online web-based data-capture system. A blood sample was taken from all participants, and further upper gastrointestinal endoscopy was performed. All biological material was delivered to Riga, Latvia, where further analysis of samples was carried out. 

*Participants*

To be eligible for participation in the study, participants had to meet the following inclusion criteria – age 40 to 64 years and no alarm symptoms potentially indicating the possibility of gastric cancer. 

In turn, exclusion criteria were – personal history of gastric or colorectal cancer, gastric resection due to benign disease, self-reported or documented *H. pylori *eradication therapy in the past, antibiotic use within one month prior to the enrolment, proton pump inhibitors or bismuth-containing drug use within two weeks prior enrolment, presence of alarm symptoms for digestive or any other diseases. 

*Laboratory methods*

Blood samples were obtained following an overnight fast. After the serum sample was received from the clinical laboratory, it was stored at -80^o^C until the testing was conducted.

All of the blood samples that were taken in the study were tested for pepsinogen I (PGI) and pepsinogen II (PGII) levels. PGI and PGII detection in plasma involved Eiken (Eiken Chemical Co., Ltd., Japan) pepsinogen test systems, which are based on the principle of latex agglutination in an autoanalyzer. Based on the Japanese experience and recommendations, we used two cut-off values: PGR ≤ 2 and PGI ≤ 30 ng/mL for severe atrophy and PGR ≤ 3 and PGI ≤ 70 ng/mL for moderate atrophy (Dinis-Ribeiro et al., 2004). 

*Upper endoscopy and biopsy samples*

Five biopsies (two from the corpus, two from the antrum, one from the incisura angularis) were taken during upper gastrointestinal endoscopy and processed according to updated Sydney System as a routine (Dixon et al., 1996). 

All biopsies were stored in 10% formalin in separate vials, and histological assessment of atrophy was based on modified Giemsa staining. All samples were assessed and reported independently via centralized data managing system by two expert gastrointestinal pathologists.

According to Sydney System (Dixon et al., 1996), the results of the biopsy, it was important to determine the prevalence of atrophy of the gastric mucosa, the corresponding moderate or severe atrophy.

*Statistics*

The analysis of the data obtained in this study was performed by the SPSS software version 22 (SPSS, Chicago, IL). The Microsoft Office Excel 2016 was used to create schedules. The descriptive statistical method for characterizing research group was used – the distribution of participants was expressed in absolute numbers and percentages. Mean age, median age, mode and standard deviation were calculated. 

From inferential statistics, T-test (to determine if there is a significant difference between the means of two group) and Chi-Square test (to analyze categorical data) were used. The level of statistical significance was set at p<0,05.

Pepsinogen serological test positive predictive value (PPV), negative predictive value (NPV) sensitivity, specificity and overall accuracy were assessed using the histological diagnosis as the “gold standard”.

*Ethical approval*

This study protocol was developed as a subordinate to the general GISTAR protocol, and its relevant amendments being approved by Ethics Committee of Riga East University Hospital Support Foundation - 03/10/2013, reg. No. 14-A/13 and the Central Medical Ethics Committee in Latvia - 09/12/2013, reg. No. 01-29.1/11.

GISTAR study has been included as a potential area of collaboration in the bilateral governmental agreement between Latvia and Kazakhstan. All individuals participating in the study signed an informed consent form.

## Results

*Participants sample characteristics*

The study included a total of 157 participants – 63 female (40,1%) and 94 male (59,9%). Participant age ranged from 40 to 64, mean age – 51 (SD – 6,98; median – 51,00; mode – 46). The difference in age composition between the gender groups was not statistically significant (p=0,399). 

*Serological pepsinogen analysis*

The mean serum PGI for male participants was 64,4 ± 3,0 ng/mL, but for females – 49,2 ± 3,1 ng/mL; mean PGII level for males was 18,5 ± 1,2 ng/mL, for females - 16,4 ± 1,2 ng/mL; mean PGR (pepsinogen I and pepsinogen II ratio) level for males was 3,4 ± 0,2 ng/mL, for females - 3,4 ± 0,2 ng/mL. The male participants had significantly higher PGI level than females (p<0,001). However, no significant difference between genders was observed for PGII and PGR levels.

*Serological pepsinogen analysis prevalence of serological atrophy*

Moderate atrophy (PGR ≤ 3 and PGI ≤ 70 ng/mL) was observed in 42 (26,8%) cases, from those 15 were corresponding to – severe atrophy (PGR ≤ 2 and PGI ≤ 30 ng/mL). Out of the 42 participants with decreased pepsinogen levels 19 (45,2%) were females, and 23 (54,8%) were males. The prevalence of decreased pepsinogen level did not differ significantly between males and females (p=0.111). 

*Histological prevalence of atrophy*

The distribution of atrophy of the gastric mucosa in certain parts of the stomach is summarized in Table 1. It should be noted that no severe atrophy was observed in the corpus and antrum part, but only one study subject had severe atrophy at the incisura angularis. Panatrophy was observed in two (1,3%) of 157 subjects.

*Accuracy of the pepsinogen test*

Further, 42 cases with PGR ≤ 3 and PGI ≤ 70 ng/mL and 15 with PGR ≤ 2 and PGI ≤ 30 ng/mL were used to analyse sensitivity, specificity, PPV and NPV of against histology. The overall accuracy of the PG test in relation to histology as a gold standard is shown in Table 2.

## Discussion

In 2018 the incidence of gastric cancer worldwide amounted to 1 033 701 cases and ranked fifth among all newly diagnosed cancers, as well as gastric cancer, was the third most common cause of tumour death worldwide in 2018 (Bray et al., 2018). 

Our group was the first which screened asymptomatic individuals in Kazakhstan, which is a high-risk area of gastric cancer development. The strength of the study is the design, i.e. endoscopic investigations with a thorough biopsy evaluation were performed for all the study subjects. Additionally, the study population included generally healthy people, and the proportion of the nationalities corresponds closely to the general population of this country. Since the incidence of gastric cancer is high in Kazakhstan, one would also expect a high prevalence of the precancerous lesions. 

Several risk factors could contribute to the high incidence of gastric cancer in Kazakhstan, like health-detrimental behaviour and poor nutrition, including a high content of salt and smoking, among others. For example, we should not forget that genetic factors play a significant role in the development of gastric cancer. For Kazakhs, it is a specific genotype (IL-1β-511T/-31C-IL1-RN2) that increases the risk of developing gastritis when interacting with *H. pylori* infection. This genotype has been identified as a high-risk factor of the development of gastric cancer in the Kazakh population (Kulmambetova et al., 2014). 

It has been reported that in certain geographical areas of Kazakhstan, such as northern Kazakhstan, the prevalence of gastritis is 18% per 100,000 populations (Kulmambetova et al., 2014). Previously, a study was conducted in Kazakhstan, where the prevalence of atrophic gastritis (determined by GastroPanel) was low – 14,1%. However, it is essential to emphasize that in the study, atrophic gastritis was detected in patients with dyspeptic complaints (Benberin et al., 2013). Our group results showed an even lower result compared to the above study - only two samples corresponded to moderate atrophy in gastric corpus. 

When analysing study subjects with decreased PG levels, no significant changes were observed between demographic factors and PG levels. No statistically significant difference was found between the gender in our study and another study from Kazakhstan (Benberin et al., 2013). The study conducted in Latvia proved the opposite result: decreased PG levels which correspond to any gastric mucosal atrophy were statistically significantly more common in women, however, similarly to this study, no statistical difference was observed between progressive atrophy and gender (Leja et al., 2012).

No studies have been previously conducted in Kazakhstan to determine the accuracy of the serological PG test for the detection of gastric mucosa atrophy. The results of our study showed that using latex agglutination method - Eiken test with the cut off PGI ≤ 70 ng/mL and PGR ≤ 3 - sensitivity was 50,0%, specificity was 73,55%, and overall accuracy was 73,25%. However, lower cut off - PGI ≤ 30 ng/mL and PGR ≤ 2 showed a higher test accuracy, it was 90,45%, but sensitivity was 50,0%, and specificity was 90,97%. Despite the relatively high accuracy of both PG values, PG test sensitivity was only 50,0% in both cases, but it should be noted that for non-invasive screening method, this result is acceptable (Huang et al., 2015). 

Our study results showed low positive predictive value for both pepsinogen cut-off values (2.3%, 6.7%). The Japanese meta-analysis also confirmed that the positive predictive value of the pepsinogens varied from 0.77% - 1.25% in population studies, while the negative predictive value is from 99.08% - 99.90% (Miki, 2006). In China, the results of the pepsinogen test have low specificity (21.2%), and the positive predictive value also was low (Gao et al., 2017). Researchers have noted that if we use gastrin-17 for gastric pre-cancerous lesions detection, then the positive predictive value can be improved; still, it will not be able to change the specificity and sensitivity (Gao et al., 2017). 

However, pepsinogen test will not be able to replace the “gold standard” - histological investigation of the atrophic gastritis.

Nevertheless, our results showed that both PG values had a high specificity, which indicates that it is possible to avoid an invasive, expensive test, such as endoscopy and use this test for detection of gastric mucosa atrophy. However, it is important to emphasize that the plasma PG test was determined in two positive individuals, and therefore the results are not reliable enough. In the future, it would be useful to investigate a larger study population to evaluate the accuracy of the test fully. In any case, for the population of Kazakhstan, we would recommend using PGI ≤ 30 ng/mL and PGR ≤ 2 values. 

As mentioned before, in this study, we used the latex agglutination method. The study from Latvia proved that the accuracy of plasma PG test differs from the threshold levels recommended by the manufacturer (Leja et al., 2017). The total accuracy of the test with cut off values - PGI ≤ 70 ng/mL and PGR ≤ 3 was 69,3%, sensitivity was 76,0%, specificity was 68,9%. Alternative cut off values - PGI ≤ 30 ng/mL and PGR ≤ 2 demonstrated accuracy 87,5%, sensitivity - 52,0%, specificity 89,9% (Leja et al., 2017). 

It should be noted that the combination of plasma PG biomarkers also showed good results in the detection of gastric cancer in China: sensitivity – 62,1%, specificity – 94,2% (Zhang et al., 2014). 

In Xinjiang, where a large Kazakh population is living, the significance of the PG test in plasma for the diagnosis of gastric cancer was determined – in particular, only Kazakh population was analyzed. The researchers found that the PG test had high specificity of 80,5% and sensitivity – 89,9%. The most useful cut off PGI for gastric cancer was ≤ 64 ng/ml and PGR ≤ 4,5 (Cai et al., 2017). 

In conclusion, the prevalence of gastric mucosal atrophy among asymptomatic individuals in Kazakhstan was very low, although the gastric cancer incidence and *H. pylori *prevalence are high in this area. The finding suggests that factors other than atrophy have a role in the gastric cancerogenesis. The diagnostic accuracy of the PG test depends on the cut-off values and the study population. Despite the knowledge of carcinogenesis of gastric cancer and the role of *H. pylori*in its development, the symptoms of gastric cancer are non-specific; therefore, an accurate screening would provide an initial advantage for high-risk patients.

In the future, it will be useful to carry out screening studies in regions with high risk for gastric cancer and Kazakhstan area could be a suitable site for this. 
